# Trends in Ambulatory Prescribing of Antiplatelet Therapy among US Ischemic Stroke Patients: 2000–2007

**DOI:** 10.1155/2012/846163

**Published:** 2012-12-05

**Authors:** Sudeep Karve, Deborah Levine, Eric Seiber, Milap Nahata, Rajesh Balkrishnan

**Affiliations:** ^1^College of Pharmacy, The Ohio State University, Columbus, OH 43210, USA; ^2^RTI Health Solutions, Research Triangle Park, Durham, NC 27709, USA; ^3^Department of Internal Medicine, College of Medicine, The Ohio State University, Columbus, OH, USA; ^4^Division of Health Services Management and Policy, College of Public Health, The Ohio State University, Columbus, OH 43210, USA; ^5^Ann Arbor VA Healthcare System and Departments of Medicine and Neurology, The University of Michigan, Ann Arbor, MI 48109, USA; ^6^Health Services Management and Policy, The Ohio State University, Columbus, OH 43210, USA; ^7^Department of Clinical, Social and Administrative Sciences, University of Michigan, Ann Arbor, MI 48109, USA; ^8^Clinical, Social and Administrative Sciences, College of Pharmacy, University of Michigan, 428 Church Street, Ann Arbor, MI 48109, USA

## Abstract

*Objective*. Study objectives were to assess temporal trends and identify patient- and practice-level predictors of the prescription of antiplatelet medications in a national sample of ischemic stroke (IS) patients seeking ambulatory care. *Methods*. IS-related outpatient visits by adults were identified using the National Ambulatory Medical Care Survey and National Hospital Ambulatory Medical Care Survey for the years 2000–2007. We assessed prescribing of antiplatelet medications using the generic drug code and drug entry codes in these data. Temporal trends in antiplatelet prescribing were assessed using the Cochran-Mantel-Haenszel test for trend. *Results*. We identified 9.5 million IS-related ambulatory visits. Antiplatelet medications were prescribed at 35.5% of visits. Physician office prescribing of the clopidogrel-aspirin combination increased significantly from 0.5% in 2000 to 22.0% in 2007 (*P* = 0.05), whereas prescribing of aspirin decreased from 17.9% to 7.0% (*P* = 0.50) during the same period. *Conclusion*. We observed a continued increase in prescription of the aspirin-clopidogrel combination from 2000 to 2007. Clinical trial evidence suggests that the aspirin-clopidogrel combination does not provide any additional benefit compared with clopidogrel alone; however, our study findings indicate that even with lack of adequate clinical evidence physician prescribing of this combination has increased in real-world community settings.

## 1. Introduction

In 2008, approximately 7 million individuals were reported to have a history of stroke [1]. Stroke survivors have a 4 to 14% annual risk of recurrent stroke and a 1–5% annual risk of myocardial infarction (MI). To reduce the recurrence of ischemic stroke (IS), the major stroke type accounting for 85% of strokes, modification of vascular risk factors [2–4], and antithrombotic therapy are recommended for stroke survivors [5, 6]. Antithrombotic therapy may include vitamin K antagonist therapy if atrial fibrillation is present (cardioembolic strokes) or antiplatelet therapy (noncardioembolic strokes). Antiplatelet therapy can reduce the relative risk of IS by approximately 15% [7]. Four antiplatelet agents (aspirin, clopidogrel, ticlopidine, and dipyridamole) are used alone or in combination to treat IS patients. However, few clinical trials of IS patients provide direct comparisons among antiplatelet alternatives. As a result, clinicians have uncertainty regarding the selection of antiplatelet therapy for secondary stroke prevention among patients with noncardioembolic IS [8–17].

Between 2001 and 2006, several clinical trials were published that may influence clinicians' prescribing of anti-platelet therapy to IS patients: CURE (Clopidogrel in Unstable Angina to Prevent Recurrent Events) [18], CREDO (Clopidogrel for the Reduction of Events During Observation trial) [19], and MATCH (Management of ATherothrombosis with Clopidogrel in High-risk patients with Recent Transient Ischemic Attacks or Ischemic Stroke) [20]. The CURE trial which showed that the combination of clopidogrel and aspirin was more effective than aspirin in reducing cardiovascular events in patients with coronary heart disease (absolute risk reduction 2%) may have led to increases in prescription of the clopidogrel-aspirin combination to patients with other types of vascular disease such as IS. Subsequently, this enthusiasm may have dampened when later trials (MATCH and CHARISMA) showed that dual antiplatelet therapy with aspirin and clopidogrel was no more effective than clopidogrel therapy alone and, in fact, the combination may be harmful. Alternatively, the European/Australasian Stroke Prevention in Reversible Ischaemia trial (ESPRIT) showed that, in patients with nonembolic recent minor cerebral ischemia, aspirin plus dipyridamole was more effective than aspirin alone in preventing vascular events, findings consistent with other studies. In 2006, updated clinical practice guidelines for secondary stroke prevention were published that generated much of these trial data and also discouraged the routine use of the aspirin and clopidogrel combination in IS patients.

Despite these major changes in the evidence and recommendations for antiplatelet therapy in vascular patients, few studies have examined temporal trends in physicians' prescribing of antiplatelet therapy to IS stroke patients in the ambulatory setting or in a population-based fashion [12]. As a result, little is known about temporal changes in ambulatory prescribing practices of antiplatelet agents to the US population of stroke survivors. In addition, the patient and physician predictors of antiplatelet therapy selection are unknown. Therefore, we assessed the temporal trends in prescribing of the various antiplatelet agents, alone or in combination, among patients with IS seeking ambulatory care using a nationally representative population-based survey. We also explored patient-level and physician-level characteristics associated with prescription of specific antiplatelet therapies. 

## 2. Materials and Methods

### 2.1. Data Source

This study was a retrospective analysis of two national surveys: (1) National Ambulatory Medical Care Survey (NAMCS) [21] and (2) National Hospital Ambulatory Medical Care Survey (NHAMCS) [22] for the years 2000 through 2007. The NAMCS is a national survey on the utilization of ambulatory medical care services provided by nonfederally employed physicians. The NHAMCS is a national survey on ambulatory care services provided in general and short stay (average length of stay <30) hospital outpatient departments (OPD) and emergency departments (ED). Federal, military, and veterans administration hospitals are excluded from this survey. Both NAMCS and NHAMCS are conducted annually and they utilize a multi-stage probability sampling with counties, groups of counties, county equivalents or towns, and townships within the US and the District of Columbia as the primary sampling unit. Both surveys provide information related to patient demographic characteristics, patient described reason for visit, physician diagnosis, payment source for the visit, and physician office/hospital location. Additionally, information on medication provided/prescribed (2000–2002: up to 6 medications are recorded; 2003–2007: up to 8 medications are recorded) during the visit is available. Each record in the data represents a patient visit. Patient visit weights provided in these data were used to obtain national estimates on ambulatory utilization at the physician office, hospital OPD, and hospital ED. 

### 2.2. Patient Population

 We identified all patients age 18 years or older who had an ambulatory visit with a primary physician diagnosis of ischemic stroke (IS) using valid ICD-9-CM codes (433.x1, 434.xx, 436.xx) [23–26] and recorded in the NAMCS and NHAMCS data from January 2000 through December 2007. Patients with a diagnosis for atrial fibrillation (ICD-9-CM code = 427.3x) or prescription for warfarin (generic drug code: 56205, drug entry codes: 34775, 07930; multum code: d00022) were excluded because these patients are likely to have cardioembolic stroke. The antiplatelet agents are primarily recommended for patients with noncardioembolic stroke and thus we excluded IS patients with diagnosis of atrial fibrillation or drug mention of warfarin.

### 2.3. Outcome Measure

The primary outcome was an ambulatory IS visit with medication mention for antiplatelet agents. The antiplatelet agents considered for this study were aspirin, clopidogrel, dipyridamole, and ticlopidine as these agents were available during 2000 to 2007. We then categorized the antiplatelet agents into the following mutually exclusive categories: (1) aspirin only, (2) clopidogrel only, (3) aspirin and clopidogrel, (4) aspirin and dipyridamole, (5) dipyridamole only, and (6) ticlopidine.

The NAMCS [21] and NHAMCS [22] collect data on medications ordered or supplied at the physician office or ED/OPD visit. The medication data is then classified and coded using the drug coding system developed by the National Center for Health Statistics (NCHS) and is made available in the NAMCS and NHAMCS dataset. To assess the proportion of IS visits resulting in an antiplatelet medication documentation we used antiplatelet drug codes provided in the NAMCS and NHAMCS data. 

### 2.4. Covariates

The various patient characteristics considered in the analysis included race (white, black, and other), age (18–44 years, 45–64 years, and ≥65 years), gender (male and female), and primary payment source for the visit (private, Medicare, Medicaid and other). The various physician office/hospital characteristics in the analysis included region of the physician office or the hospital as defined by the US census bureau (Northeast, Midwest, South, West), location (urban: Metropolitan Statistical Area (MSA), rural: non-MSA), and visit setting (physician office, hospital OPD, and hospital ED).

## 3. Statistical Analysis

 Patient visit weights were used to assess the national estimate on annual IS visits with and without a mention of antiplatelet agents. Temporal changes in the proportion of IS visits resulting in mention of antiplatelet agents were assessed during the 8-year study period, that is, 2000 to 2007, using the Cochran-Mantel-Haenszel test for trend. We further stratified the utilization trends by visit setting, that is, physician office, hospital ED, and hospital OPD, using indicator variable for visit setting available in the dataset. Associations between antiplatelet prescribing and patient and physician office/hospital characteristics were tested using the Chi-square test. All the statistical analyses were performed in SAS-callable SUDAAN (version 10.0.1 hosted on the Windows platform) to account for the complex survey design of the NAMCS and NHAMCS and to provide weighted results that reflect population estimates. This study was approved by the Institutional Review Board at the Ohio State University. 

## 4. Results and Discussion

### 4.1. Results

#### 4.1.1. Patient Characteristics

 During the 8 year period, there were 9.5 million ischemic stroke-related ambulatory visits of which 6.8 million (71.1%) occurred in a physician office, 0.3 million (3.4%) in a hospital OPD, and 2.4 million (25.5%) in a hospital ED (Table 1). Over 77% of the visits were by whites and approximately 50.1% were by females; 67.5% were by patients aged over 65 years. Among persons aged <65 years the proportion of visits increased from 26.8% in 2000-01 to 36.7% in 2006-07, whereas, the proportion of visits in the MSA region increased from 71.1% in 2000-01 to 92.4% in 2006-07. 

#### 4.1.2. Associations between Patient and Physician Office/Hospital Characteristics and Antiplatelet Medications Prescribed

Table 2 represents the univariate association between patient and physician office/hospital characteristics and antiplatelet drugs prescribed. No significant differences in prescribing of aspirin or clopidogrel monotherapy or aspirin-clopidogrel combination were observed by race or region. Older adults were more likely to receive clopidogrel monotherapy compared with younger adults (*P* = 0.03). The aspirin-clopidogrel combination was significantly more likely to be prescribed among men compared with women (80.3% versus 19.7%; *P* < 0.01). Clopidogrel monotherapy (88.7%, *P* < 0.01) or in combination with aspirin (89.0%, *P* = 0.01) was more likely to be prescribed in a physician office compared with a hospital ED or a OPD. Overall, 0.98 million visits resulted in prescribing of aspirin-clopidogrel combination, for which the proportion increased significantly from 2.4% (0.02 million) in 2000-01 to 31.2% (0.30 million) in 2006-07 (*P* < 0.01). 

#### 4.1.3. Antiplatelet Prescribing Trend

**Figure 1:**
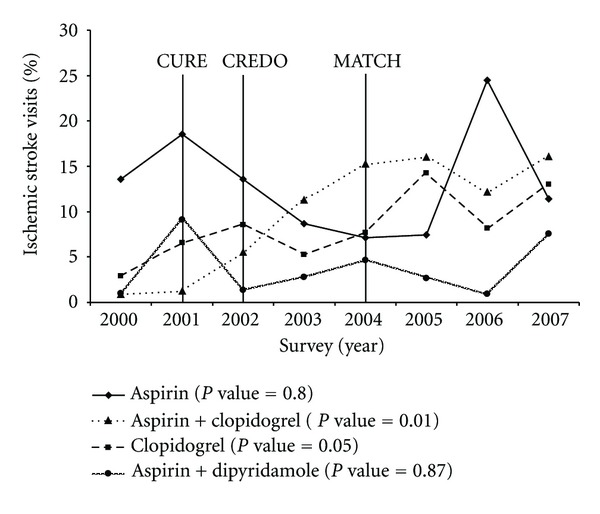
Antiplatelet prescribing trends among patients with ischemic stroke: NAMCS, NHAMCS, 2000–2007. NAMCS: National Ambulatory Medical Care Survey; NHAMCS: National Hospital Ambulatory Medical Care Survey; *P* values based on Cochran-Mantel-Haenszel test for trend. Years of publication of the 3 clinical trials shown in the figure above: 2001—CURE (Clopidogrel in Unstable Angina to Prevent Recurrent Events trial); 2002—CREDO (Clopidogrel for the Reduction of Events During Observation); 2004—MATCH (Management of ATherothrombosis with Clopidogrel in High-risk patients).

Among the IS patients, the proportion of patients receiving antiplatelet drugs increased from 28.1% in 2000-01 to 47.1% in 2006-07 (*P*_trend_ = 0.02) (Table 1). In 2000, the proportion of visits resulting in clopidogrel-aspirin combination was 0.8% which significantly increased to 16.1% in 2007 (*P*_trend_ = 0.01) (Figure 1). 

No significant changes in prescribing of dipyridamole-aspirin combination were seen (*P*_trend_ = 0.87) in this study. During the same period, the proportion of patients receiving aspirin monotherapy declined from 13.6% in 2000 to 11.4% in 2007 (*P*_trend_ = 0.80), while prescribing for clopidogrel monotherapy increased significantly from 2.9% in 2000 to 13.0% in 2007 (*P*_trend_ = 0.05). 

**Figure 2:**
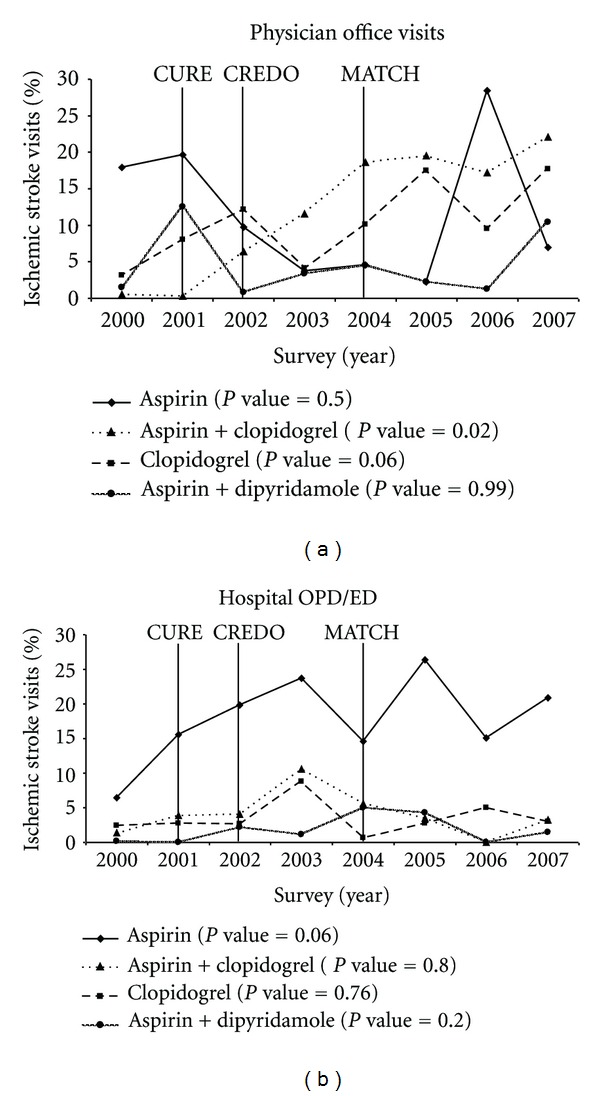
Antiplatelet prescribing trends among patients with ischemic stroke, by visit setting: 2000–2007. Physician office visits based on National Ambulatory Medical Care Survey data; Hospital ED/OPD visits based on National Hospital Ambulatory Medical Care Survey data; OPD: Outpatient Department; ED: Emergency Department; *P* values based on Cochran-Mantel-Haenszel test for trend. Years of publication of the 3 clinical trials shown in the figure above: 2001—CURE (Clopidogrel in Unstable Angina to Prevent Recurrent Events trial); 2002—CREDO (Clopidogrel for the Reduction of Events During Observation); 2004—MATCH (Management of ATherothrombosis with Clopidogrel in High-risk patients).

Prescribing trends varied significantly by the physician practice setting (Figure 2). Proportion of IS patients receiving clopidogrel-aspirin combination significantly increased in the physician office setting (2000: 0.5%–2007: 22.0%; *P*_trend_ = 0.02) whereas no significant changes in prescribing were observed in the hospital OPD/ED (2000: 1.4%–2007: 3.2%; *P*_trend_ = 0.81) (Figure 2). In contrast, the proportion of IS patients receiving aspirin decreased considerably in the physician office setting (2000: 17.9%–2007: 7.0%; *P*_trend_ = 0.50), while it increased significantly in the hospital OPD/ED setting (2000: 6.5%–2007: 20.9%; *P*_trend_ = 0.06) (Figure 2). No significant differences in prescribing of clopidogrel monotherapy and dipyridamole-aspirin combination in either setting were observed.

## 5. Discussion

 To the authors knowledge this is the first comprehensive study evaluating the ambulatory (physician office, hospital ED, and hospital OPD) prescribing trends for antiplatelet agents among community dwelling IS patients. We have identified significant changes in utilization pattern of antiplatelet agents among IS patients. During the 8 year study period (2000–2007), clopidogrel-aspirin prescribing increased significantly in the physician office setting. In contrast, the prescribing of clopidogrel-aspirin combination remained relatively low and stable in the hospital OPD and ED during the same period. However, the prescription of aspirin monotherapy increased dramatically in the hospital OPD and ED settings while it declined significantly in the physician office setting. We found that prescribing of clopidogrel alone was considerably higher among elderly compared with younger IS patients. Prior study findings suggest that the risk of bleeding is higher among elderly patients using aspirin plus clopidogrel combination compared with patients only using clopidogrel [27]. Additionally, a study assessing risk factors associated with bleeding reported that compared with aspirin only users, patients using aspirin in combination with ticlopidine or clopidogrel had a 68% higher risk of bleeding (odds ratio: 1.68; 95% confidence interval: 1.02–1.77) [20]. We also found a higher use of clopidogrel plus aspirin combination among males compared with females. Findings of a recent meta-analysis suggest that the use of clopidogrel plus aspirin was associated with lower risk of CVD events among both men and women; however, the addition of clopidogrel to aspirin therapy was associated with a 43% and 21% increased risk of bleeding among females and males, respectively [28].

 Our findings suggest that physician prescribing of clopidogrel-aspirin combination may have been influenced by the publication of three major clinical trials. Findings from both CURE (08/01) [18] and CREDO (11/02) [19] suggest that the use of clopidogrel-aspirin combination can significantly reduce the relative risk for the primary outcome measure (death from cardiovascular causes, nonfatal myocardial infarction, or IS). Publication of these trials along with aggressive marketing and promotion by the drug manufacturers [12] may have resulted in the increased prescribing of the clopidogrel-aspirin combination during the study period. 

During the period under consideration for this study, MATCH [20] was the third major published trial evaluating clopidogrel-aspirin combination versus aspirin monotherapy for the primary composite endpoint; that is, ischemic stroke, myocardial infarction, vascular death, or rehospitalization for acute ischemia. The findings of this study did not indicate any benefit of adding aspirin to clopidogrel treatment in reducing the risk of the primary outcome but in contrast increased the risk of bleeding. Hill and Johnston reported a decline in hospital use of this combination following the publication of MATCH [12, 20], in contrast we found that the use of this combination continued to increase especially in the physician office setting after publication of MATCH. However, we did observe an increase in the prescribing of clopidogrel monotherapy in physician office setting following the publication of MATCH. Similarly, the American Heart Association/American Stroke Association guidelines published in 2006 that cautioned on the use of aspirin-clopidogrel combination did not seem to have an effect on physician office prescribing of this combination [6].

We found a significant increase in prescribing of aspirin monotherapy in the hospital OPD and ED settings from 2000 to 2007. One of the reasons for this increased use may be the publication of the Chinese Acute Stroke Trial [29] and International Stroke Trial [30] and subsequent publication of the American Stroke Association guidelines (07/02) [31], recommending the use of aspirin among patients suspected with acute stroke. Moreover since patients visiting hospital ED were more likely to present with acute IS it may explain the increased use of aspirin monotherapy in hospital ED setting. 

Surprisingly, during the entire study period the prescribing of dipyridamole-aspirin combination remained low even though this combination was shown to be effective in reducing the risk of recurrent IS or death as compared to aspirin monotherapy in the European Stroke Prevention Study 2 (ESPS-2) published in 1996 [32]. However, we did observe a considerable increase in prescribing of this combination in 2007. This increase may be due to the publication of ESPRIT trial in 2006, which highlighted the relative risk reduction in the primary outcome measure (i.e., composite of death from all vascular causes, nonfatal stroke, nonfatal myocardial infarction, or major bleeding complication) among patients using dipyridamole-aspirin combination compared with patients using aspirin monotherapy [33].

In the wake of the above findings, there is a need to recognize certain limitations of this study. In this study the proportion of patients receiving antiplatelet medications ranged from 28% to 47% compared to 89% reported by Hill and Johnston [12]. This may be due to several reasons; firstly, both the NAMCS and NHAMCS data do not provide information on several important factors such as stroke severity, stroke type (first versus recurrent, acute versus nonacute), and contraindication to antiplatelet therapy. Secondly, antiplatelet agents are primarily recommended for patients with noncardioembolic IS; the data we used does not permit distinction between patients with cardioembolic or noncardioembolic stroke. However, we excluded patients with atrial fibrillation and those using warfarin considering this as a proxy for patients with cardioembolic stroke. Moreover, the important distinction between these two studies is the setting; our study focused on the ambulatory prescribing trends which may vary significantly from inpatient prescribing. Additionally, our analysis was restricted to patients with primary IS diagnosis selected using previously validated high sensitivity and specificity ICD-9-CM codes (433.x1, 434.xx, and 436.xx). Expanding the analysis to patients with secondary IS diagnosis or the use of other low sensitivity ICD-9-CM code(s) (e.g., 433.xx—without the 5th digit modifier) may affect the national estimates on IS-related outpatient visits and anti-platelet prescribing trends. In sensitivity analyses using a low-specificity algorithm (433.xx, 434.xx, and 436.xx) [34, 35], estimates on the number of IS-related ambulatory visits varied; however the proportion of patients receiving antiplatelet therapy remained relatively similar (data available upon request). The cross-sectional nature of the survey does not permit the evaluation of a patient's prior IS treatment. Even though the data provide information on both prescription and over the counter (OTC) medications prescribed or provided at the visit, the OTC availability of aspirin may underestimate the actual reporting of the aspirin use. Moreover, for both NAMCS and NHAMCS data the information on maximum number of medications provided has changed over the years; information on 6 medications was available during 2000 to 2002 which increased to 8 medications in 2003 to 2007. Finally, NHAMCS data do not contain information on physician specialty and thus we could not assess the association between physician specialty and antiplatelet use.

## 6. Conclusions

Our study highlights important changes in the prescribing patterns of antiplatelet therapy among IS patients. Our findings suggest that even with the lack of adequate efficacy evidence, safety concerns, and higher cost, the prescribing of clopidogrel-aspirin combination increased substantially during the study period. Quality improvement measures are warranted to educate physicians of the evidence regarding antiplatelet drugs for secondary stroke prevention and improve prescribing of safe antiplatelet drugs among IS patients. 

## Figures and Tables

**Table 1:** Ischemic stroke patient characteristics and antiplatelet agents prescribed: NAMCS, NAMCS 2000–2007^a,b^.

Characteristic	Survey Year							
	2000-01		2002-03		2004-05		2006-07	
	(*N* = 285)		(*N* = 330)		(*N* = 352)		(*N* = 304)	
	Population estimate	%	Population estimate	%	Population estimate	%	Population estimate	%
Total ischemic stroke visits	**2,221,277**	**100%**	**2,238,540**	**100%**	**2,917,650**	**100%**	**2,150,851**	** 100%**

Race								
White	1,605,408	72.3%	1,760,410	78.6%	2,266,603	77.7%	1,716,210	79.8%
Black	448,739	20.2%	184,064	8.2%	551,036	18.9%	392,536	18.3%
Other	167,130	7.5%	294,066	13.1%	100,011	3.4%	42,105	2.0%

Region								
Northeast	340,302	15.3%	509,922	22.8%	393,472	13.5%	339,938	15.8%
Midwest	491,280	22.1%	366,347	16.4%	1,058,706	36.3%	536,408	24.9%
South	703,364	31.7%	579,150	25.9%	1,119,719	38.4%	928,226	43.2%
West	686,331	30.9%	783,121	35.0%	345,753	11.9%	346,279	16.1%

Age								
18–44 years	46,907	2.1%	141,937	6.3%	137,665	4.7%	116,652	5.4%
45–64 years	549,189	24.7%	536,338	24.0%	885,617	30.4%	673,635	31.3%
≥65 years	1,625,181	73.2%	1,560,265	69.7%	1,894,368	64.9%	1,360,564	63.3%

Gender								
Female	1,177,727	53.0%	1,196,813	53.5%	1,572,905	53.9%	834,076	38.8%
Male	1,043,550	47.0%	1,041,727	46.5%	1,344,745	46.1%	1,316,775	61.2%

Insurance^c^								
Private insurance	523,035	23.5%	553,314	24.7%	860,712	29.5%	633,122	29.4%
Medicare	1,488,002	67.0%	1,364,738	61.0%	1,538,343	52.7%	1,224,087	56.9%
Medicaid	76,721	3.5%	61,621	2.8%	106,824	3.7%	128,987	6.0%
Other	90,939	4.1%	206,476	9.2%	145,752	5.0%	75,490	3.5%

Location								
MSA^d^	1,580,138	71.1%	2,003,578	89.5%	2,546,768	87.3%	1,986,970	92.4%
Non-MSA	641,139	28.9%	234,962	10.5%	370,882	12.7%	163,881	7.6%

Antiplatelet prescribing								
Overall antiplatelet agents	623,998	28.1%	641,900	28.7%	1,101,786	37.8%	1,010,462	47.0%
Aspirin only	360,067	16.2%	244,855	10.9%	213,034	7.3%	377,002	17.5%
Clopidogrel only	107,873	4.9%	151,764	6.8%	327,148	11.2%	231,652	10.8%
Aspirin and clopidogrel	23,542	1.1%	192,779	8.6%	456,787	15.7%	305,702	14.2%
Aspirin and dipyridamole	118,183	5.3%	47,861	2.1%	104,817	3.6%	96,106	4.5%
Dipyridamole only	3,148	0.1%	0	0.0%	0	0.0%	0	0.0%
Ticlopidine only	11,185	0.5%	4,641	0.2%	0	0.0%	0	0.0%

Setting								
Physician office	1,502,370	67.6%	1,550,687	69.3%	2,231,054	76.5%	1,493,699	69.4%
Hospital OPD	65,065	2.9%	102,196	4.6%	62,847	2.2%	92,825	4.3%
Hospital ED	653,842	29.4%	585,657	26.2%	623,749	21.4%	564,327	26.2%

^a^NAMCS: National Ambulatory Medical Care Survey, NHAMCS: National Hospital Ambulatory Medical Care Survey.

^b^Population estimates were calculated using SAS-callable SUDAAN software, version 10.0.1 (Research Triangle Institute) to obtain proper variance estimations that accounted for the complex sampling design of the National Ambulatory Medical Care Survey and National Hospital Ambulatory Medical Care Survey and results that were weighted to reflect national population estimates.

^c^Insurance (payment source) does not sum to 100% because of missing data, ^d^MSA: Metropolitan Statistical Area.

**Table 2:** Association between patient demographic and physician office/hospital characteristics and antiplatelet agent prescribed: NAMCS, NHAMCS 2000–2007^a , b^.

	Antiplatelet medication											
Characteristic	Aspirin only			Clopidogrel only			Aspirin and clopidogrel			Aspirin and dipyridamole		
	(*N* = 233)			(*N* = 95)			(*N* = 63)			(*N* = 39)		
	Population estimate	%	*P* value	Population estimate	%	*P* value	Population estimate	%	*P* value	Population estimate	%	*P* value

Total	1,194,958	—		818,437	—		978,810	—		366,967	—	

Race												
White	958,151	80.2%	0.40	581,489	71.0%	0.68	708,008	72.3%	0.91	341,339	93.0%	0.11
Black	201,745	16.9%		150,228	18.4%		203,509	20.8%		22,867	6.2%	
Other	35,062	2.9%		86,720	10.6%		67,293	6.9%		2761	0.8%	

Region												
Northeast	304,874	25.5%	0.38	82,604	10.1%	0.52	111,832	11.4%	0.47	62,510	17.0%	0.75
Midwest	217,812	18.2%		287,327	35.1%		161,843	16.5%		108,731	29.6%	
South	433,594	36.3%		259,449	31.7%		466,195	47.6%		84,482	23.0%	
West	238,678	20.0%		189,057	23.1%		238,940	24.4%		111,244	30.3%	

Age												
18–44 years	65,453	5.5%	0.39	10,958	1.3%	**0.03** ****	17,561	1.8%	0.20	12364	3.4%	0.90
45–64 years	436,552	36.5%		116,735	14.3%		404,818	41.4%		95,313	26.0%	
≥65 years	692,953	58.0%		690,744	84.4%		556,431	56.8%		259,290	70.7%	

Gender												
Female	632,517	52.9%	0.71	522,671	63.9%	0.15	192,540	19.7%	**<0.01**	113,778	31.0%	0.22
Male	562,441	47.1%		295,766	36.1%		786,270	80.3%		253,189	69.0%	

Insurance^c^												
Private insurance	355,686	29.8%	0.31	187,508	22.9%	0.47	405,119	41.4%	0.21	64,802	17.7%	0.73
Medicare	656,260	54.9%		572,429	69.9%		461,148	47.1%		267,898	73.0%	
Medicaid	71,523	6.0%		26,884	3.3%		53,025	5.4%		10,678	2.9%	
Other	55,345	4.6%		21,151	2.6%		14,927	1.5%		10,620	2.9%	

Location												
MSA^d^	994,903	83.3%	0.82	646,403	79.0%	0.58	852,053	87.0%	0.72	224,322	61.1%	0.30
Non-MSA	200,055	16.7%		172,034	21.0%		126,757	13.0%		142,645	38.9%	

Setting												
Physician office	711,071	59.5%	**0.05** ****	726,058	88.7%	**<0.01**	870,856	89.0%	**0.01** ****	317,518	86.5%	0.21
Hospital OPD^e^	56,218	4.7%		49,195	6.0%		5,305	0.5%		8,951	2.4%	
Hospital ED	427,669	35.8%		43,184	5.3%		102,649	10.5%		40,498	11.0%	

Year												
2000-01	360,067	30.1%	0.13	107,873	13.2%	0.22	23,542	2.4%	**<0.01**	118,183	32.2%	0.62
2002-03	244,855	20.5%		151,764	18.5%		192,779	19.7%		47,861	13.0%	
2004-05	213,034	17.8%		327,148	40.0%		456,787	46.7%		104,817	28.6%	
2006-07	377,002	31.5%		231,652	28.3%		305,702	31.2%		96,106	26.2%	

^a^NAMCS: National Ambulatory Medical Care Survey, NHAMCS: National Hospital Ambulatory Medical Care Survey.

^b^Population estimates were calculated using SAS-callable SUDAAN software, version 9.0.1 (Research Triangle Institute) to obtain proper variance estimations that accounted for the complex sampling design of the National Ambulatory Medical Care Survey and National Hospital Ambulatory Medical Care Survey and results that were weighted to reflect national population estimates.

^c^Insurance (payment source) does not sum to 100% because of missing data, ^d^MSA: Metropolitan Statistical Area.

^e^OPD: Outpatient Department.
